# The influence of early serum heparin-binding protein (HBP) levels on the occurrence of sepsis in patients with severe burns

**DOI:** 10.5937/jomb0-59279

**Published:** 2026-02-27

**Authors:** Lihan Xiang, Guimei Yang, Liming Lou, Le Lv, Liyuan Zheng, Caixia Zhang, Qian He

**Affiliations:** 1 Zhejiang Chinese Medical University, Hangzhou City, China; 2 Department of Critical Care Medicine, Ezhou Central Hospital, Ezhou City, China; 3 Department of Pulmonary and Critical Care Medicine, the Third Affiliated Hospital of Zhejiang Chinese Medical University, Hangzhou City, China; 4 Department of Hepatobiliary and Pancreatic Surgery, the Second Affiliated Hospital of Zhejiang University School of Medicine, Hangzhou, Zhejiang Province, China; 5 Department of Day Care, The Eighth Medical Centre, Chinese PLA General Hospital, Beijing, China; 6 Department of General Surgery, The Eighth Medical Centre, Chinese PLA General Hospital, Beijing, China

**Keywords:** heparin-binding protein (HBP), severe burn, sepsis, early warning index, survival prognosis, heparin-vezujući protein (HBP), teške opekotine, sepsa, indeks ranog upozorenja, prognoza preživljavanja

## Abstract

**Background:**

To evaluate the expression levels of early serum heparin-binding protein (HBP) in patients with severe burns and investigate its prognostic significance and predictive utility for sepsis development.

**Methods:**

The clinical data of 52 patients with severe burns who were admitted to our hospital between January 2023 and May 2025 were retrospectively analysed. General information, including the patient's age, sex, body mass index (BMI), total burn area, and tracheal intubation or tracheotomy, was collected. Within 48 hours of the patient's admission, the patient's blood pressure, white blood cell count, serum procalcitonin (PCT), and serum C-reactive protein (CRP) level were measured. The second assessment of acute physiology and chronic health within 48 hours of admission included the results of the sequential organ failure assessment (SOFA) and APACHE II. The patients were separated into two groups based on their conditions at discharge: the nonsurviving group and the surviving group.

**Results:**

Compared with the nonsepsis group (8.2 ng/mL), the sepsis group had a considerably greater median early serum HBP level (20.8 ng/mL) (P&lt;0.001). The area under the curve (AUC) of HBP for sepsis prediction, according to the ROC curve analysis, was 0.87 (95% CI: 0.82-0.92). The sensitivity was 85.0%, and the specificity was 82.1% at the cutoff value of 14.6 ng/mL. After controlling for confounding variables such as burn area and inhalation injury, multivariate logistic regression analysis verified that HBP&gt;14.6 ng/mL was an independent risk factor for the development of sepsis (OR=4.53, 95% CI: 2.67-7.69). Survival analysis revealed that patients in the high-HBP group had a significantly greater 28-day mortality rate (log-rank P&lt;0.01). Patients in the death group were older than those in the survival group, and this difference was statistically significant (P=0.036). The area under the ROC curve (AUC) of the HBP level for predicting patient death during hospitalization was 0.798; its sensitivity and specificity were 88.33% and 70.00%, respectively, for HBP 147.03 ng/mL. In patients with sepsis following severe burns, the AUC of the HBP level for the prediction of septic shock was 0.789. Its sensitivity and specificity were 90.00% and 63.20%, respectively, when the HBP concentration was greater than 147.03 ng/mL.

**Conclusions:**

The significantly elevated serum HBP level in patients with severe burns in the early stage is closely related to the risk of sepsis, and it can be used as a sensitive biomarker for early warning of sepsis.

## Abstract (sr)

**Uvod:**

Procena nivoa ranog serumskog heparin-vezujuceg proteina (HBP) kod pacijenata sa teškim opekotinama i ispitivanje njegovog prognostičkog značaja i značaja u prognozi za razvoj sepse.

**Metode:**

Retrospektivno su analizirani klinički podaci 52 pacijenta sa teškim opekotinama, primljenih u našu bolnicu u periodu od januara 2023. do maja 2025. godine. Prikupljeni su opšti podaci, uključujući starost, pol, indeks telesne mase (BMI), ukupnu površinu opekotina i prisustvo trahealne intubacije ili traheotomije. Tokom prvih 48 sati od prijema, mereni su krvni pritisak, broj leukocita, nivo serumskog prokalcitonina (PCT) i C-reaktivnog proteina (CRP). U istom periodu izvršene su i procene prema SOFA i APACHE II skorovima. Pacijenti su podeljeni u dve grupe na osnovu ishoda pri otpustu: preživeli i preminuli.

**Rezultati:**

Medijana ranog nivoa serumskog HBP bila je značajno viša u grupi sa sepsom (20,8 ng/mL) u poređenju sa grupom bez sepse (8,2 ng/mL) (P&lt;0,001). HBP je predvideo smrt tokom hospitalizacije sa AUC vrednošću od 0,798, senzitivnošću od 88,33% i specifičnošću od 70,00% za HBP 147,03 ng/mL.

**Zaključak:**

Značajno povišen serumski nivo HBP u ranoj fazi kod pacijenata sa teškim opekotinama blisko je povezan sa rizikom od sepse i može se koristiti kao osetljiv biomarker za rano upozorenje na sepsu.

## Introduction

Patients with severe burns have damaged skin defence barriers, continuous invasion of pathogenic microorganisms, disordered immune functions, and the presence of necrotic tissue on the wound surface, which can easily lead to systemic infections and even sepsis [1]. At present, sepsis has become the primary cause of poor prognosis in patients with severe burns [2][3]. Once sequential multisystem dysfunction occurs in patients with sepsis, it is often difficult to correct. Therefore, early warning, timely diagnosis and intervention are the keys to the successful treatment of patients with severe burns. Infection biochemical markers, such as serum procalcitonin (PCT) and serum C-reactive protein (CRP), have specific auxiliary diagnostic value. Still, there are problems such as low specificity and long reaction times.

Heparin-binding protein (HBP), an acute-phase protein, has attracted much attention for its early warning value in the occurrence and development of severe infections and sepsis in patients in the intensive care unit [4][5]. However, the early warning value of this indicator for the prognosis of patients with severe burns has rarely been reported.

This study retrospectively analysed the early warning value of HBP expression levels within 48 hours after severe burns for patient death and the occurrence of sepsis. It explored the correlation between this indicator and the prognosis of patients with sepsis, aiming to provide a basis for early diagnosis, timely intervention of sepsis in patients with severe burns, and improvement of the poor prognosis of patients with severe burns.

## Materials and methods

### Research subjects

A retrospective analysis of the clinical data of patients with severe burns admitted to our hospital from January 2023 to May 2025 was conducted.

Inclusion criteria: (1) Aged 18-98 years; (2) The total burn area is 50% of the total body surface area (TBSA). The total burn area is comprehensively measured and calculated via the Chinese nine-part method for the burn area and the palm method; (3) The depth of the burn is deep degrees II to III; (4) The cause of the burn is hot liquid or flame burns, and the patient was admitted to the hospital within 8 hours after the burn.

Exclusion criteria: (1) Death within 72 hours after burn; (2) Burns accompanied by craniocerebral trauma, abdominal organ injury, blast injury, inhalation injury, fractures, etc., or combined with chemical burns and severely contaminated wounds; (3) Those who have had serious systemic diseases in the past, such as heart, lung, liver, kidney and blood system diseases, as well as malignant tumors; (4) Pregnant or lactating; (5) Those who have been using hormones or immunosuppressants for a long time; (6) Those with incomplete medical records; (7) Those who voluntarily give up treatment halfway; (8) Those who have already experienced severe hypovolemic shock and hypoxemia upon arrival at the hospital.

### Grouping of research subjects and basis

Patients were divided into two groups according to their status at discharge: the nonsurviving group and the surviving group. Patients were divided into a sepsis group and a non-sepsis group according to whether sepsis occurred during hospitalisation. Patients with sepsis were divided into a sepsis non-shock group and a sepsis shock group according to whether shock occurred. The diagnostic criteria for burn sepsis adopted the Sepsis 3.0 criteria [5]. They referred to the diagnostic guidelines for burn sepsis proposed by the Burn Physicians Branch of the Medical Doctor Association in 2020 [6]. The diagnostic criterion for shock in burn sepsis [7] is persistent hypotension based on a clear diagnosis of sepsis. However, based on adequate blood volume supplementation, vasopressor drugs are still needed to maintain a mean arterial pressure of 65 mmHg (1 mmHg = 0.133 kPa) and a serum lactate level of >2 mmol/L.

### Treatment methods and data collection

After admission, all patients routinely underwent effective fluid resuscitation and airway management and were given wound and systemic anti-infection measures, nutritional support, and organ function protection and support treatments. Wound treatment includes dressing changes, staged and batched scab excision and skin grafting surgeries, etc.

The general information of the patients, such as sex, age, body mass index (BMI), total burn area, injury factors, tracheal intubation or tracheotomy, etc., was collected. The acute physiology and chronic health evaluation II (acute physiology and chronic health evaluation II) results of the patients were recorded within 48 hours after admission, and indicators such as the APACHE II score; sequential organ failure assessment (SOFA) score; PCT, CRP and HBP levels; white blood cell (WBC) count; and microbial culture results were recorded. All the clinical laboratory samples were sent to the Clinical Laboratory Department of Ruijin Hospital Affiliated with Shanghai Jiao Tong University School of Medicine for testing.

The normal reference values of each index are PCT<0.5 μg/L, CRP 0-10 mg/L, WBC (4-10)x10^9^/L, and HBP<11.4 ng/mL. The APACHE II score [8] consists of three parts: the acute physiology score (APS), age score, and chronic health score (CPS), and the final score is the sum of the three scores. Its theoretical maximum score is 71 points. The higher the score is, the more severe the condition. The SOFA score [9] consists of the functional scores of six major organs or systems: the respiratory system, the coagulation system, the digestive system (Liver function), the circulatory system, the nervous system, and the urinary system (Kidney function). The higher the score is, the more severe the degree of organ/system dysfunction or failure.

### Statistical analysis

Data description and statistical analysis were performed via SPSS 26.0 software. The quantitative data with a normal distribution are expressed as x±s and were analysed via the independent sample t-test. Quantitative data with a nonnormal distribution are expressed as M (Q1, Q3) and were analysed via the Wilcoxon rank sum test. Qualitative data are expressed as frequencies (percentages) and were analysed via the χ^2^ test and Fisher's exact probability method.

Logistic regression was used to analyse the influencing factors. The variables with statistical significance in the univariate analysis were included in the multivariate analysis to establish a prediction model, and the receiver operating characteristic curve (ROC curve) was plotted. P<0.05 indicated that the difference was statistically significant.

## Results

### General condition of the patient

A total of 52 patients were included, among whom 46 were male and 6 were female. The average age was 47±14 years, the average BMI was 23.72±3.31 kg/m^2^, the proportion of total burn area in the TBSA was 76.80±13.25%, and 37 patients (71.15%) underwent tracheotomy or tracheal intubation treatment.

A comparison of the general conditions of the patients in the different groups revealed that age was significantly related to survival (P=0.036), that the total burn area and whether tracheal intubation or tracheotomy was performed were significantly different according to whether sepsis occurred in patients with severe burns (all P<0.05), and that there were no statistically significant differences in the other data (Table 1, Table 2, Table 3).

**Table 1:** Comparison of the general situation of death and survival groups.

Item	Death group (n=12)	Survival group (n=40)	t/χ^2^ value	P value
Gender (%)	—	—	0.402	0.526
Male	10 (83.33)	36 (90.00)		
Female	2 (16.67)	4 (10.00)		
Age (Year)	54.17±15.25	44.43±13.25	-2.158	0.036
Percentage of TBSA (%)	82.50±10.66	71.55±14.08	-2.482	0.165
Endotracheal intubation or tracheotomy (%)	11 (91.67)	26 (65.00)	3.198	0.074
BMI (kg-m^-2^)	22.42±3.42	24.20±3.15	1.68	0.098

**Table 2:** Comparison of the general situation of patients with and without sepsis.

Item	Nonseptic shock group<br>(n=19)	Septic shock group<br>(n=10 )	t/χ^2^ value	P value
Gender (%)			3.372	0.105
Male	18 (94.74)	7 (70.00)		
Female	1 (5.26)	3 (30.00)		
Age (Year)	47.26±14.94	48.30±10.25	0.196	0.846
Percentage of TBSA (%)	79.79±10.78	85.30±8.08	1.416	0.168
Endotracheal intubation or tracheotomy (%)	17 (89.47)	9 (90.00)	0.002	0.965
BMI (kg-m^-2^)	23.98±3.45	22.87±3.46	-0.826	0.416

**Table 3:** Comparison of the general situation of patients with and without septic shock.

Item	Sepsis group (n=29)	Nonsepsis group (n=2 )	t/Z/χ^2^ value	P value
Gender (%)			0.327	0.568
Male	25 (86.21)	21 (91.30)		
Female	4 (13.79)	2 (8.70)		
Age (Year)	47.62±13.32	44.43±13.25	-0.537	0.554
Percentage of TBSA (%)	85.00 (75.00, 89.50)	61.00 (53.00, 75.00)	2.393	0.011
Endotracheal intubation or tracheotomy (%)	26 (89.66)	11 (47.83)	10.93	0.001
BMI (kg-m^-2^)	23.60±3.43	24.04±3.12	0.481	0.636
Prognosis (%)			0.751	0.386
Survival	21 (72.41)	19 (82.61)		
Death	8 (27.59)	4 (17.39)		

### Comparison of laboratory indicators and APACHE II and SOFA scores between the nonsurviving group and the surviving group

As shown in Table 4, the levels of HBP PCT, and CRP and the WBC count within 48 hours after admission were greater in the nonsurviving group than in the surviving group. Among them, the difference in HBP between the two groups was statistically significant (P=0.002), whereas there was no statistically significant difference in other indicators. In addition, compared with those in the survival group, the APACHE II scores of patients in the death group within 48 hours of admission were significantly greater (P=0.032). There was no statistically significant difference in the SOFA score between the two groups.

**Table 4:** Comparison of laboratory results, APACHE II and SOFA scores in different prognosis groups of patients.

Item	Death group (n=12)	Survival group (n=40)	t/χ^2^ value	P value
HBP (ng-mL^-1^)	201.92 (156.12, 260.82)	101.96 (20.02, 168.90)	3.306	0.002
WBC (x10^9^-L^-1^)	22 .96±8.72	21.81±10.14	0.354	0.725
CRP (mg-L^-1^)	108.47 (3.35, 212.60)	65.78 (6.15, 108.28)	1.705	0.094
PCT (mg-L^-1^)	4.50 (1.43, 6.78)	4.47 (0.50, 5.45)	0.01	0.286
APACHE II/score	18.50±2.65	17.00±1.87	2.208	0.032
SOFA/score	15.33±2.64	14.98±1.85	0.532	0.597

### Comparison of laboratory indicators and APACHE II and SOFA scores between the sepsis group and the nonsepsis group

As shown in Table 5, compared with that in the nonsepsis group, the HBP level within 48 hours of admission in the sepsis group was significantly greater (P=0.014), whereas there was no statistically significant difference in other laboratory indicators. In addition, the APACHE II and SOFA scores within 48 hours of admission in the sepsis group were significantly greater than those in the nonsepsis group (all P<0.05). Compared with that of the sepsis without shock group [114.36 (19.47, 169.24)], the HBP level of patients within 48 hours of admission was significantly greater in the sepsis shock group [202.77 (71.58, 300.00)] (P=0.008).

**Table 5:** Comparison of laboratory results, APACHE II and SOFA scores in different groups of patients with and without sepsis.

Item	Sepsis group (n=29)	Nonsepsis group (n=23)	t/χ^2^ value	P value
HBP (ng-mL^-1^)	144.84 (38.27, 258.40)	75.13 (21.36, 98.70)	2.556	0.014
WBC (x10^9^-L^-1^)	24.07±9.85	19.55±9.26	1.688	0.098
CRP (mg-L^-1^)	83.99 (11.30, 135.25)	65.09 (4.90, 116.40)	0.872	0.68
PCT (mg-L^-1^)	5.12 (0.79, 6.32)	3.05 (0.52, 1.17)	1.078	0.286
APACHE II/score	18.21±2.16	16.26±1.57	3.621	0.032
SOFA/score	15.86±1.86	14.04±1.80	3.549	0.001

### Logistic regression was used to analyse the risk factors for death and septic shock in patients with severe burns

Whether the patients died during hospitalisation was taken as the outcome indicator and included HBP CRP and PCT levels and WBC count for logistic regression analysis. The results (Table 6) revealed that the HBP level within 48 hours after admission (OR=1.011, 95% CI 1.001-1.095) was related to death. Whether septic shock occurred was taken as the outcome indicator and included in the above indicators for logistic regression analysis. The results (Table 7) revealed that the HBP level at 48 hours after admission (OR=1.013, 95% CI 0.001-2.064) was related to whether septic shock occurred.

**Table 6:** Logistic regression analysis of the risk factors affecting the death of severely burned patients.

Variable	β	SE	P value	OR	95%CI
WBC	-0.003	0.045	0.944	0.997	-0.122-0.109
HBP	0.011	0.004	0.013	1.011	1.001-1.095
CRP	0.004	0.005	0.46	1.004	-0.015-0.190
PCT	-0.041	0.064	0.518	0.96	-0.240-0.148

**Table 7:** Logistic regression analysis of risk factors affecting the occurrence of septic shock in severely burned patients with sepsis.

Variable	β	SE	P value	OR	95%CI
WBC	0.003	0.064	0.965	1.003	-1.735 -6.315
HBP	0.013	0.006	0.048	1.013	0.001-2.064
CRP	0.013	0.009	0.149	1.013	-0.013-1.575
PCT	0.033	0.071	0.645	0.968	-12.644-2.125

### The early warning value of HBP level for the risk of death in patients with severe burns

**Figure 1:**
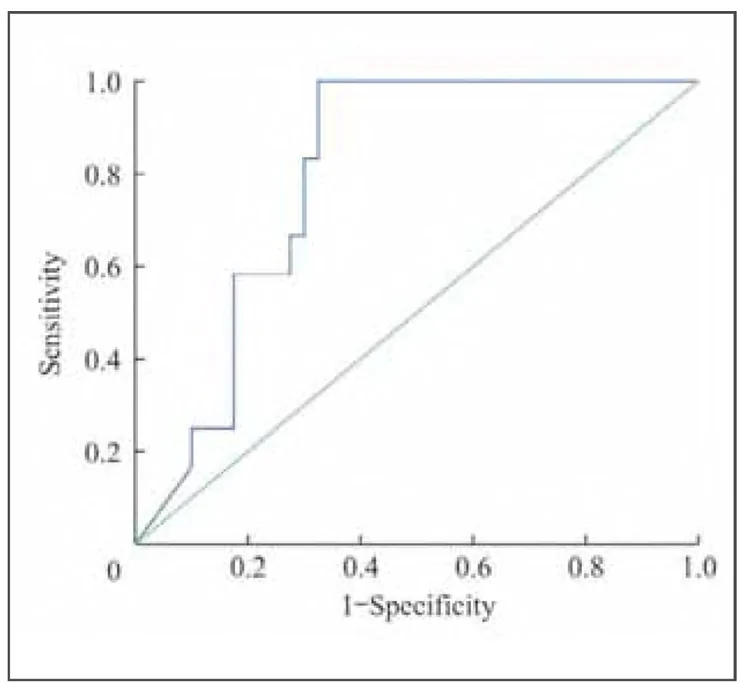
ROC curve for HBP in the mortality risk of severely burned patients.

The ROC curve (Figure 1) revealed that the area under the curve (AUC) of the HBP level for predicting death in patients with severe burns was 0.798 (95% CI 0.681-0.915, P=0.002); when HBP 147.03 ng/mL, its sensitivity and specificity were 88.33% and 70.00%, respectively.

### The early warning value of HBP for septic shock in patients with severe burn sepsis

**Figure 2:**
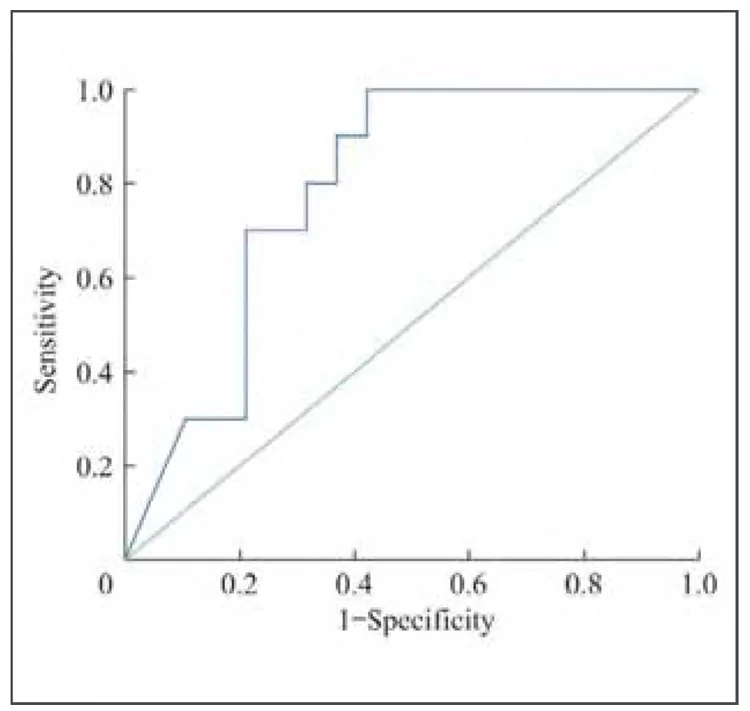
ROC curve for HBP in septic shock patients with severe burns.

The ROC curve (Figure 2) revealed that the AUC of the HBP level for the prediction of septic shock in patients with severe burn sepsis was 0.789 (95% CI 0.626-0.953, P=0.012); when the HBP level was 147.03 ng/mL, its sensitivity and specificity were 90.00% and 63.20%, respectively.

## Discussion

Sepsis is a life-threatening form of organ dysfunction caused by dysregulation of the body's response to infection. Burns have unique pathophysiological characteristics. Most burn patients experience two peaks of infection. One is the oedema recovery period (3 to 7 days after burn), and the other occurs 2 to 3 weeks after burn. Sepsis is one of the most common complications in patients with severe burns; it is often caused by multidrug-resistant bacteria and is very difficult to treat. According to statistics, the incidence rate of burn sepsis in China is as high as 43.9% [6]. Once a patient develops sepsis, the waterfall effect formed by inflammatory mediators and immune factors in the body leads to systemic inflammatory response syndrome and irreversible multiorgan function damage and can cause septic shock. The mortality rate of patients with burn sepsis combined with septic shock is as high as 70% [7]. Therefore, early diagnosis, timely intervention and dynamic assessment are the key measures to reduce the occurrence of sepsis in patients with severe burns and reduce their poor prognosis. Given that severe infections in patients with severe burns usually occur 3 days to 3 weeks after injury, it is necessary to »move forward« the assessment time point [8][9][10]. However, within 48 hours after injury, the burn shock stage occurs, and signs of infection are not fully »revealed« or »masked« by the clinical manifestations of the shock stage, such as hypovolemia. Traditional indicators such as body temperature, neutrophil percentage, WBC count, and platelet count lack specificity [11][12]. At this time, bacteriological tests are often negative, making simple clinical assessment lacking a strong basis.

Serum biochemical markers are clinical indicators that have been developed in recent years and are beneficial for infection assessment. They can help physicians quickly determine the existence of infection and infer the types of possible infectious pathogens. The commonly used biochemical markers at present include CRP PCT, and interleukin-6 (IL-6), which have specific early warning values in the diagnosis and treatment of infectious diseases. However, the specificity of indicators such as CRP and IL-6 is relatively poor [13], and changes in these indicators are more affected by factors such as surgery and trauma. PCT is a glycoprotein without hormone activity, and it is positively correlated with the severity of bacterial infection. It increases rapidly 2 to 4 hours after infection and reaches its peak 12 to 48 hours later. PCT has relatively high specificity and is recommended by numerous guidelines and consensuses at home and abroad [14][15][16]. However, it is strongly affected by noninfectious factors and has certain limitations, especially in the early stage after burn injury. Positive blood culture is another criterion for the clinical diagnosis of sepsis [17][18][19]. However, the waiting time for culture results is long, the positive rate of early specimen culture is low, and false negatives are prone to occur, which cannot achieve early and rapid warning.

Previous studies [20][21][22] have shown that the HBP is early, specific and sensitive in the diagnosis of bacterial infections and sepsis. HBP is the only secretory protein present in neutrophil granules. The level of HBP in the blood of healthy people generally does not exceed 10 ng/mL. Once infected, neutrophils are activated, and secretory vesicles are rapidly excreted, releasing HBP, which can induce endothelial cells to express intercellular cell adhesion molecule-1 (ICAM-1). This enables polymorphonuclear leukocytes (PMNs) to adhere to the endothelial surface, promoting the release of HBP by the PMN. HBPs reactivate Ca^2+^ channels, allowing Ca^2+^ to flow into the cells and resulting in the rearrangement of the endothelial cytoskeleton. The rearrangement of the endothelial cytoskeleton causes cell contraction and an increase in the intercellular space, ultimately leading to an increase in endothelial cell permeability [23]. Disruption of the endothelial barrier is an important pathophysiological mechanism of sepsis. Similarly, after severe burns, endotoxins released by gram-negative bacteria induce vascular endothelial injury, resulting in destruction of the vascular barrier, increased permeability, and elevated serum HBP levels.

Furthermore, studies have shown that the HBP level in patients with sepsis is highly valuable for the rapid diagnosis of early severe bacterial infections and is an effective biomarker for evaluating the severity of the disease in patients with sepsis. It is imperative in the early diagnosis and monitoring of therapeutic effects in patients with septic shock [24][25][26]. At present, the diagnostic value of HBP has been reported in patients with urinary tract infections, severe pancreatitis, diabetic ketoacidosis, severe cases of novel coronavirus infection, etc. However, for clinical burn treatment, there are currently few reports on the detection, level analysis and effect evaluation of HBP [27][28][29].

The combined sensitivity of HBP in the diagnosis of sepsis was 0.85 (95% CI 0.79-0.90), and the combined specificity was 0.91 (95% CI 0.82-0.96), both of which were greater than those of PCT and CRP Therefore, HBP has greater value than PCT and CRP in the diagnosis of sepsis. Furthermore, an international multicenter study from Sweden, the United States and Canada collected plasma samples from 759 suspected infected patients to measure the levels of HBP PCT, CRP and lactic acid, and WBC count 12-24 hours later. Among the 487 patients, 141 developed organ dysfunction syndrome within 72 hours, and 78% of the patients had elevated plasma HBP levels (>30 ng/mL) before the occurrence of organ dysfunction syndrome. Compared with PCT, CRP, and lactic acid levels and the WBC count, HBP is the best biomarker for the diagnosis and early warning of organ dysfunction. This study revealed that, compared with that in the nonsepsis group, the HBP level in the sepsis group of patients with severe burns was greater. Moreover, the HBP level in the septic shock group was significantly greater than that in the septic nonshock group (all P<0.05). The ROC curve also indicated that HBP has a certain early warning value for adverse prognostic outcomes. The above results suggest that with the aggravation of sepsis, the level of serum HBP gradually increases, thereby leading to the aggregation of inflammatory cells and an increase in vascular permeability, aggravating damage to body tissues and subsequently causing septic shock [30]. In addition, the results of this study revealed that the HBP level of patients with severe burns in the death group was greater than that in the survival group, suggesting that HBP can predict the outcome of patients with severe burns to a certain extent. The ROC curve also confirmed that HBP has a certain early warning value for the prognosis of patients with severe burns [31].

The APACHE II and SOFA scores can reflect the severity of injury to various organs in acute and chronic diseases. The results of this study revealed a statistically significant difference in the APACHE II score between the death group and the survival group of patients with severe burns (P<0.05), and the differences in the APACHE II and SOFA scores between patients who developed sepsis were also statistically significant (all P<0.05). These findings indicate that the APACHE II and SOFA scores have certain early warning values for the prognosis of patients with severe burns. However, the data in the APACHE II and SOFA scoring systems are relatively abundant, the calculations are cumbersome, and they are vulnerable to subjective factors, resulting in overly high or low predictions of the prognostic outcomes of patients by these two scores [32]. Therefore, if one wants to obtain a relatively accurate score, it is necessary to observe the patient's condition dynamically; otherwise, deviations are prone to occur. As a simple laboratory indicator, HBP is not affected by subjective factors and has advantages in predicting the poor prognosis of patients with severe burns.

There are several limitations in this study. First, this was a single-centre retrospective study involving only patients with a single disease from a single department, and the number of included patients was relatively small. In the future, the sample size can be further expanded for exploration. Second, the time points designed for recording in this study were selected and recorded by the researchers through clinical observations. Whether they have similar early warning values at other time points requires further research. Therefore, designing a multicenter prospective study is highly valuable for obtaining a comprehensive understanding of the potential of HBP in predicting the occurrence and prognosis of sepsis in burn patients.

## Conclusion

The expression level of serum HBP within 48 hours of hospitalisation can be used to evaluate the prognosis of patients with severe burns, which is helpful for clinicians to take intervention measures as early as possible to prevent patient death and the occurrence of septic shock.

## Dodatak

### Authors' contributions

Lihan Xiang and Guimei Yang are the first co-authors of the study.

### Conflict of interest statement

All the authors declare that they have no conflict of interest in this work.
